# New perspective for evaluating the main *Camellia oleifera* cultivars in China

**DOI:** 10.1038/s41598-020-77609-7

**Published:** 2020-11-26

**Authors:** Quanen Deng, Jianan Li, Chao Gao, Junyong Cheng, Xianzhen Deng, Dezhi Jiang, Liang Li, Ping Yan

**Affiliations:** 1 Key Laboratory of Non-Wood Forest Product of State Forestry Administration, Key Laboratory of Cultivation and Protection for Non-Wood Forest Trees of Ministry of Education, 2011 Cooperative Innovation Center of Cultivation and Utilization for Non-Wood Forest Trees of Hunan Province, Engineering Technology Research Center of Southern Hilly and Mountainous Ecological Non-Wood Forest Industry of Hunan Province, College of Forestry, Central South University of Forestry and Technology, No. 498 Shaoshan South Road, Changsha, 410004 China; 2 Institute for Forest Resources & Environment of Guizhou, Guizhou University, Guiyang, 550025 China; 3 Non-Wood Forest Research Institute, Hubei Academy of Forestry, Wuhan, 430075 China; 4 Hubei Macheng Wunaoshan National Forest Park Management Office, Macheng, 438300 China

**Keywords:** Developmental biology, Evolution, Plant sciences, Environmental sciences

## Abstract

To assess the adaptability of *Camellia oleifera* for introduction in new growth locations, this study evaluated 10 representative *C. oleifera* cultivars from the main areas in China where this oil-producing evergreen crop is grown. Cluster analysis, correlation analysis, and membership function analysis were used to evaluate various indices of the selected *C. oleifera* cultivars, including flowering phenology, cold tolerance, leaf structure, pollen characteristics, and pollen viability. The correlation analysis identified the full blossoming time, leaf palisade and spongy tissue thickness, pollen deformity rate, and pollen activity as key indices for determining the adaptability of the cultivars to new areas. The membership function analysis of the 10 *C. oleifera* cultivars revealed the following order of adaptability: ‘XLC25’ > ‘Changlin4hao’ > ‘Ganzhouyou8hao’ > ‘Ganzhouyou6hao’ > ‘Tiechengyihao’ > ‘Eyou465’ > ‘XLC10’ > ‘Changlin3hao’ > ‘Changlin18hao’ > ‘QY235.’ When introducing *C. oleifera* cultivars to new regions, the higher-ranked cultivars are more likely to be successful. The results of this study may provide a new direction for the comprehensive assessment of plant introduction and domestication potential, i.e., the assessment of the vegetative and reproductive growth, adversity resistance, and blossoming time of plants.

## Introduction

The introduction of exotic plants to new areas has taken place for thousands of years and contributed substantially to the historical progression of the four ancient civilizations, i.e., ancient China, Egypt, Babylon and India^1^. It has played an inestimable role in promoting agricultural development, food availability, population growth and economic and social progress and therefore has served as a power source for human agricultural civilizations and the development of industrial civilizations.

Exotic plant introductions can be affected by a variety of factors, such as temperature, humidity and illumination. To date, numerous studies on successful exotic introduction have been reported, and useful experience has been obtained. Zhang^2^ investigated the adaptability of five *Camellia sinensis* cultivars after their introduction from low-altitude areas to high-altitude areas. He found that the germination time of the cultivars was closely associated with major climate factors, such as temperature, illumination and humidity; that introductions at multiple locations had a higher success rate than introduction at single location; and that evergreen multiple-shoot *C. sinensis* cultivars may need certain accumulated low temperatures to release their dormancy. However, most of the published studies on plant introduction have focused on beans, and studies on other plant species are rare.

**Figure 1:**
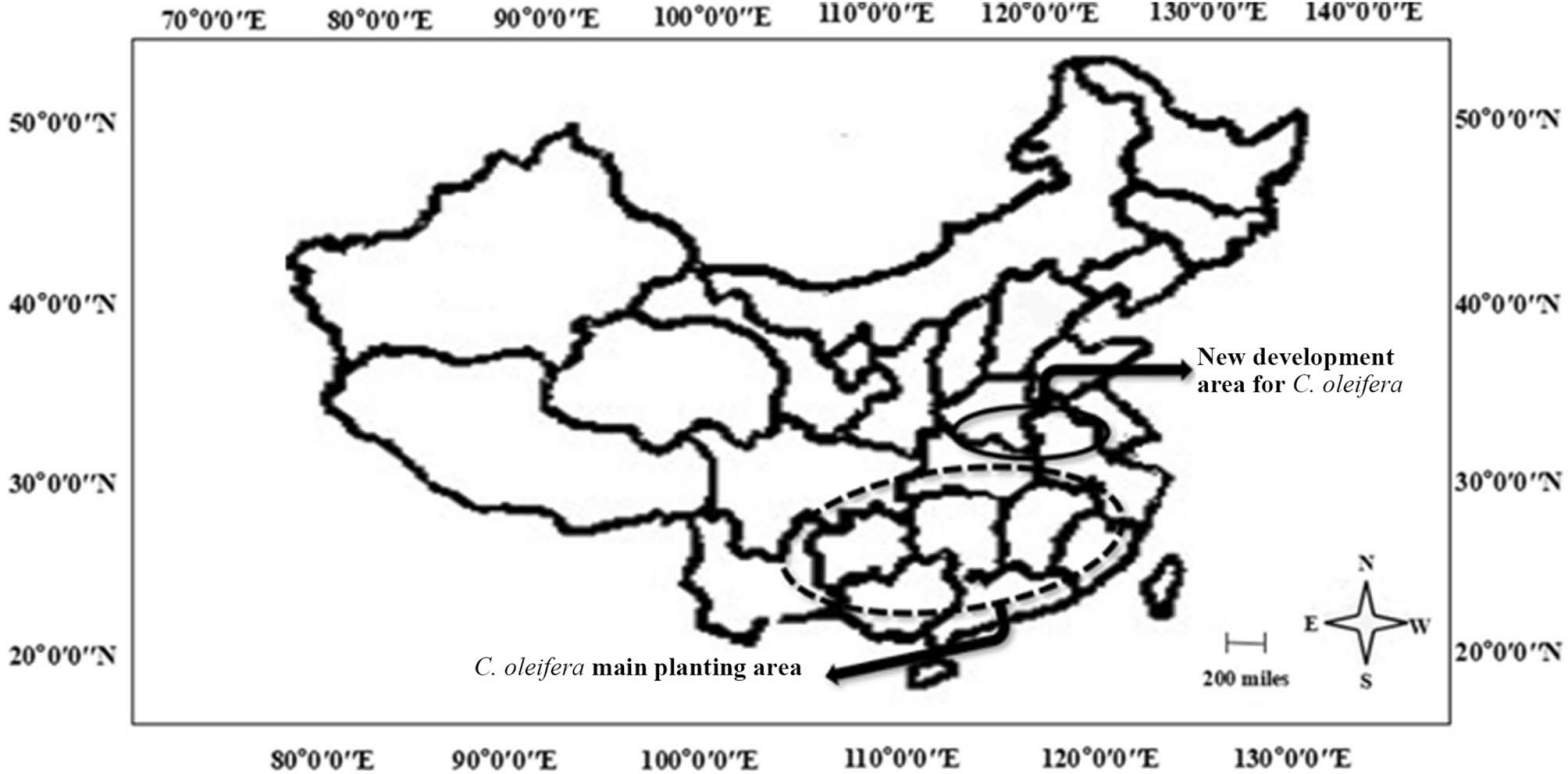
Culture distribution range of *C. oleifera* in China (plotted with AI CS6, http://b2.chengyi-ad.com.cn/1/ai/). The solid circle in the map indicates the newly developed regions for *C. oleifera,* and the dotted circle indicates the main production area (original area).

*Camellia oleifera* is the most important traditional edible-oil-producing woody species in China; it is also one of the four major woody oil trees across the world^3^. Geographically, the distribution of *C. oleifera* ranges from 23° 30′ to 31° 00′ N and from 104° 30′ to 121° 25′ E (Fig. 1). In China, *C. oleifera* undergoes flower bud differentiation between late spring and early summer and blossoms in autumn, and these processes occur at the same time as a transition from high temperature to low temperature. In recent years, global climate changes have become increasingly serious, and extreme climate events occur frequently. According to the fifth report by the Intergovernmental Panel on Climate Change (IPCC), the global average surface temperature increased by 0.85 °C from 1880 to 2012 (IPCC, 2013). In China, the warming rate is even higher, with noticeable regional and seasonal features^4^, which leads to varying degrees of change in the boundaries of climate-based natural regions^5^. These changes will inevitably affect the planting distribution of crops^6^. As a consequence, the planting distribution of *C. oleifera* in China is changing, showing a continuous northward shift, and in its originally suitable areas, *C. oleifera* production is being reduced due to extreme weather.

In compliance with Chinese policies about poverty alleviation, many new *C. oleifera* orchards have been planted in parts of Henan, Anhui, and Hubei Provinces in recent years, which cover the northern range in which *C. oleifera* can be grown in China (Fig. 1). As with other plants, the main *C. oleifera* production areas are places of interest for research as well as of excellent cultivar breeding. At the new orchards in Hubei, Anhui and Henan, there is a lack of local excellent cultivars, and these cultivars have to be introduced from Hunan and Jiangxi Provinces. However, in the main producing areas, large areas of aged and weak *C. oleifera* forests exist that started growing dozens of years ago. These forests normally have mixed cultivars with noticeable cultivar degeneration; cultivar transmission is needed in these forests. Currently, crown replacement by high grafting is the normally adopted method for cultivar transmission, and this technique inevitably involves the issue of the selection of the transferred cultivars. To date, the transfer of *C. oleifera* from the south where it is grown to the north has been reported^7^. However, most of the studies focus on the cultivar ‘Changlin’, and there is a lack of studies on the adaptability of *C. oleifera* cultivars introduced from the main producing areas. Therefore, research on the adversity resistance and growth performance of different *C. oleifera* cultivars after introduction into new regions still needs to be carried out.

Based on the aforementioned information, this study comprehensively assessed the vegetative and reproductive growth of 10 *C. oleifera* cultivars in homogenous orchards that were introduced from their main production areas. The results of this study may provide a new method of performing adaptability assessments for plant introduction, that is, performing adaptability assessments in terms of comprehensive indices, such as growth, adversity resistance and flowering phenology.

## Results

### Flowering time

The flowering time of the *C. oleifera* cultivars varied greatly, and the durations of the different flowering stages differed as well (Table 1, Columns 4 to 6). Among the investigated cultivars, ‘QY235’ showed the earliest intial blossoming stage (IBS), full blossoming stage (FuBS) and final blossoming stage (FiBS) October 14, October 18 and October 31, respectively), whereas ‘XL51’, ‘Ganzhouyou8hao’ and ‘XLC25’ showed the latest IBS, FuBS and FiBS (November 15, December 1 and December 8, respectively). The shortest and longest FBT durations were observed in ‘Cenruan2hao’ (6 days) and ‘XLC10’ (25 days), respectively. According to the correlation analysis (Table 2), FuBS had a significant positive correlation with IBS (correlation coefficient, 0.882; P < 0.05) and a very significant positive correlation with FiBS (correlation coefficient, 0.932; P < 0.01). FuBS had a significant negative correlation with the FuBS duration time (FuBSDT; correlation coefficient, − 0.688; P < 0.05). Therefore, the FuBS was the most important index for the cluster analysis.

**Table 1:** Dates and standardized data for flowering times of different *C. oleifera* varieties observed in 2017.

Serial number	Original region	Cultivar	IBS (month-day)	FuBS (month-day)	FiBS (month-day)	IBS (days)	FuBS (days)	FiBS (days)	FuBSDT (days)
1	Guangxi	‘Cenruan3hao’	11–08	11–13	11–20	21	26	21	8
2		‘Cenruan2hao’	11–15	11–27	12–02	23	40	33	6
3	Hubei	‘QY235’	**10–14**	**10–18**	**10–31**	1	1	1	14
4		‘Eyou102hao’	11–04	11–13	11–21	22	26	22	9
5		‘Eyou276hao’	10–19	11–08	11–21	6	21	22	14
6		‘Eyou361hao’	10–19	10–21	11–11	6	4	12	22
7		‘Eyou465hao’	10–30	11–02	11–16	17	15	17	15
8	Jiangxi	‘Ganwu1hao’	11–13	11–24	11–30	31	37	31	7
9		‘Ganwu2hao’	11–01	11–10	11–26	19	23	27	17
10		‘Ganwu11hao’	11–13	11–21	11–28	31	34	29	8
11		‘Ganzhouyou6hao’	11–06	11–10	11–19	24	23	20	10
12		‘Ganzhouyou8hao’	11–21	12–01	12–08	39	44	39	8
13	Hunan	‘XLC25hao’	11–15	11–27	12–08	33	40	39	12
14		‘XL51hao’	11–24	11–30	12–07	42	43	38	8
15		‘Changlin3hao’	10–17	11–09	11–23	4	22	24	15
16		‘XLC10hao’	10–21	10–24	11–17	8	7	18	25
17		‘Tiechengyihao’	10–28	11–03	11–18	15	16	19	16
18	Zhejiang	‘Changlin4hao’	11–12	11–15	11–25	30	28	26	11
29		‘Changlin22hao’	11–13	11–17	11–26	31	30	27	10
20		‘Changlin27hao’	11–04	11–11	11–25	22	24	26	15
21		‘Changlin40hao’	11–05	11–10	11–26	23	23	27	17
22		‘Changlin18hao’	10–16	10–25	11–02	3	8	3	9

The dates in bold were the reference dates based on which standardized data were obtained. IBS, FuBS, FiBS and FuBSDT represent the initial blossoming stage, the full blossoming stage, the final blossoming stage and the full blossoming stage duration, respectively.

**Table 2:** Correlations between different flowering stages based on Pearson correlation analysis.

	IBS	FuBS	FiBS	FuBDT
IBS	1			
FuBS	0.882**	1		
FiBS	0.839**	0.932**	1	
FuBDT	− 0.571*	− 0.688*	− 0.379	1

*Significant at (P = 0.05), **Significant at (P = 0.01).

IBS, FuBS, FiBS, and FuBDT represent the initial blossoming stage, the full blossoming stage, the final blossoming stage and the full blossoming stage duration, respectively.

**Figure 2:**
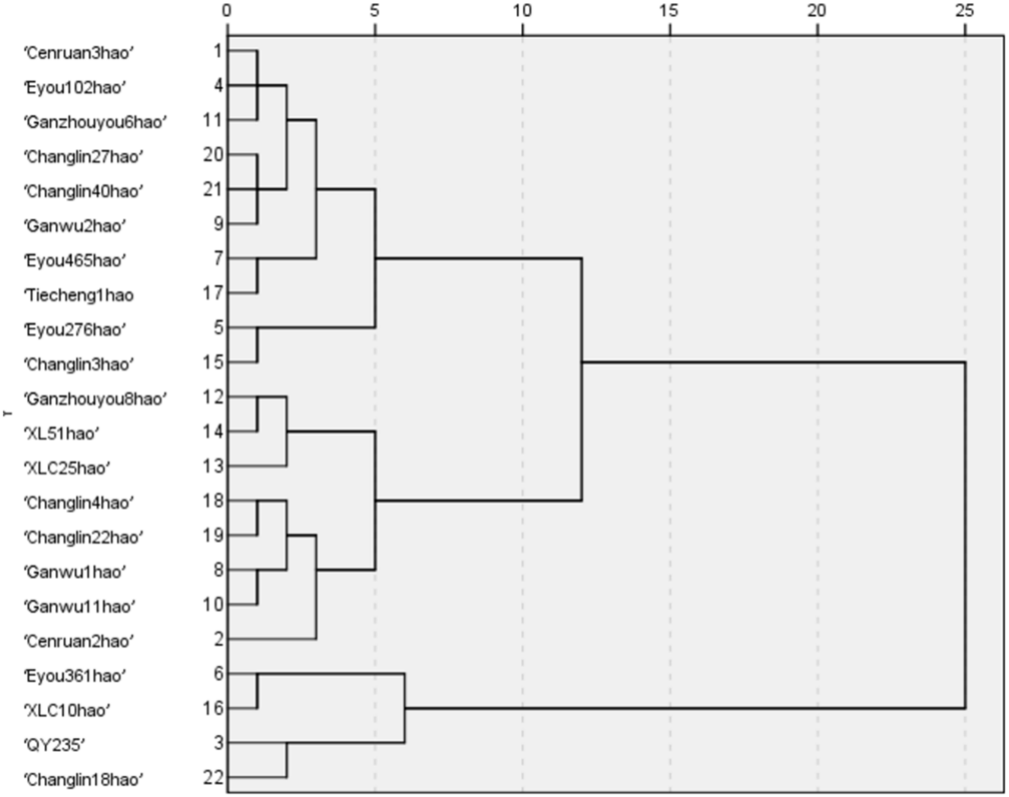
Dendrogram from the hierarchical cluster analysis of 22 *C. oleifera* based on the three blossoming stages (IBS, FuBS, FiBS) and the full blossoming stage duration time (FuBSDT).

Hierarchical cluster analysis based on IBS, FuBS, FiBS and FuBSDT separated the 22 *C. oleifera* cultivars into three groups (Fig. 2). The first group contained four early-blossoming cultivars, namely, ‘Changlin18hao’, ‘QY235’, ‘XLC10’ and ‘Eyou361’. The second group consisted of 10 midseason-blossoming cultivars, namely, ‘Cenruan2hao’, ‘Eyou102hao’, ‘Ganzhouyou6hao’, ‘Changlin27hao’, ‘Changlin40hao’, ‘Ganwu2hao’, ‘Eyou465hao’, ‘Tiechengyihao’, 'Eyou276hao' and ‘Changlin3hao’. ‘Ganzhouyou8’, ‘XL51hao’, ‘XLC25hao’, ‘Changlin4hao’, ‘Changlin22hao’, ‘Ganwu1hao’, ‘Ganwu11hao’ and ‘Cenruan2hao’ constituted the third, late-blossoming group.

### Cold tolerance

**Figure 3:**
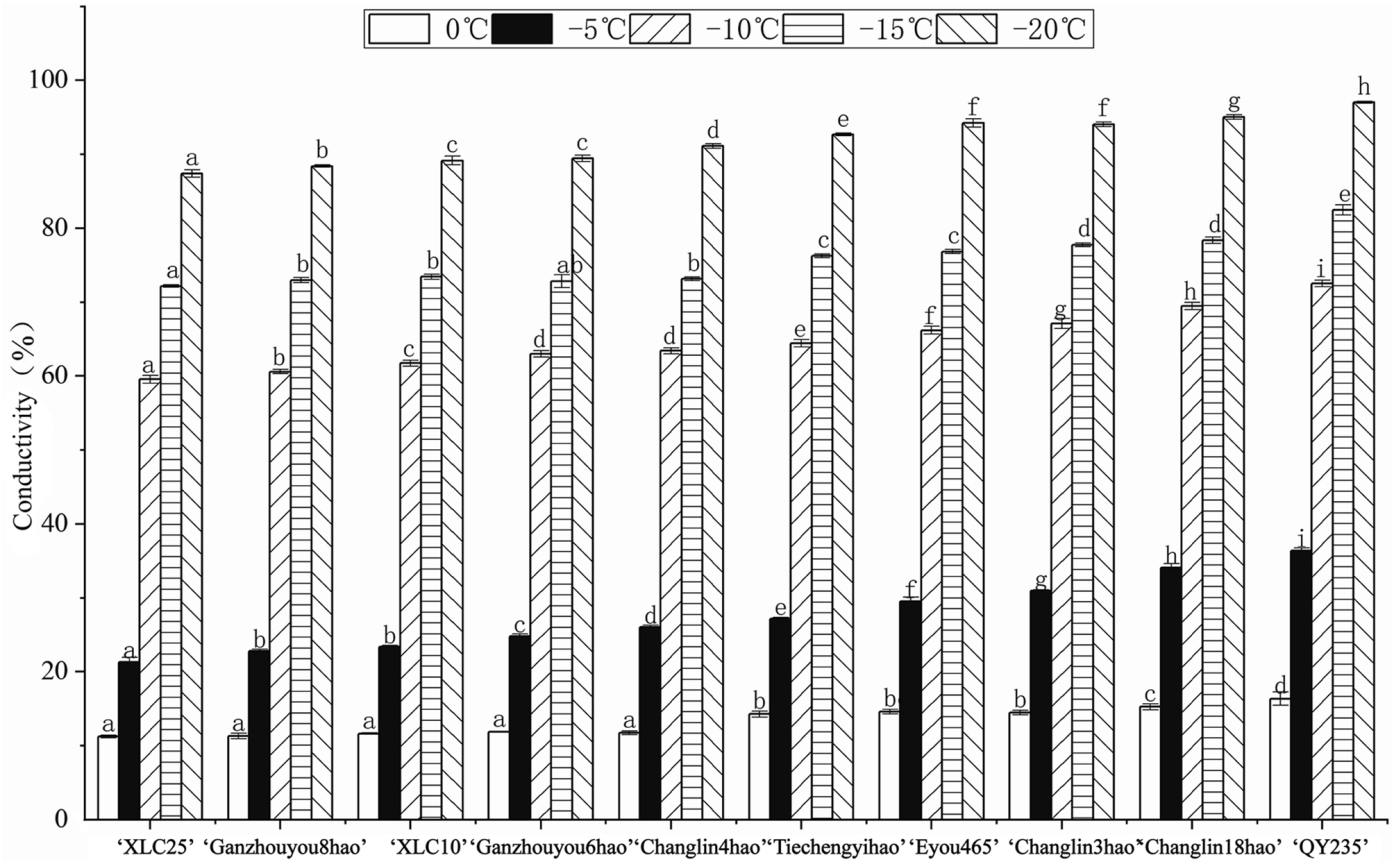
Relative conductivity of 10 *C. oleifera* cultivars under low-temperature stress. Different letters within the same temperature indicate significant differences (p < 0.05) among the 10 *C. oleifera* cultivars.

As the incubation temperature decreased, the relative conductivity of the leaves of all the 10 investigated *C. oleifera* cultivars increased (Fig. 3), with the most obvious increase observed between − 5 and − 10 °C. According to their semilethal low temperature (LT_50_) values, the cold resistance capacities of the cultivars were ranked as follows: ‘XLC25’ > ‘Ganzhouyou8hao’ > ‘XLC10’ > ‘Ganzhouyou6hao’ > ‘Changlin18hao’ = ‘Tiechengyihao’ > ‘Eyou465’ > ‘Changlin3hao’ > ‘Changlin18hao’ > ‘QY235’ (Table 3). The LT_50_ of the 10 *C. oleifera* cultivars was between − 6 and − 10 °C, which was basically consistent with the relative conductivity outcomes.

**Table 3:** The median lethal temperature for different *C. oleifera* cultivars.

Cultivar	Regression equation	LT_50_ (°C)	Correlation coefficient/R^2^
‘XLC25’	y = 96.96/(1 + 8.57e^−0.22x^)	− 9.88	0.992
‘XLC10’	y = 96.66/(1 + 7.76e^−0.21x^)	− 9.58	0.994
‘Ganzhouyou8hao’	y = 96.32/(1 + 7.20e^−0.21x^)	− 9.73	0.994
‘Ganzhouyou6hao’	y = 96.19/(1 + 7.60e^−0.21x^)	− 9.48	0.994
‘Changlin4hao’	y = 99.26/(1 + 9.73e^−0.25x^)	− 8.80	0.978
‘Changlin18hao’	y = 96.92/(1 + 5.16e^−0.21x^)	− 7.66	0.989
‘Tiechengyihao’	y = 95.29/(1 + 5.92e^−0.20x^)	− 8.80	0.991
‘Changlin3hao’	y = 96.78/(1 + 5.713e^−0.21x^)	− 8.15	0.993
‘Eyou465’	y = 96.5/(1 + 5.86e^−0.21x^)	− 8.41	0.993
‘QY235’	y = 98.21/(1 + 5.15e^−0.24x^)	− 6.96	0.990

*LT*_*50*_ semilethal low temperature.

### Leaf anatomical structure

**Figure 4:**
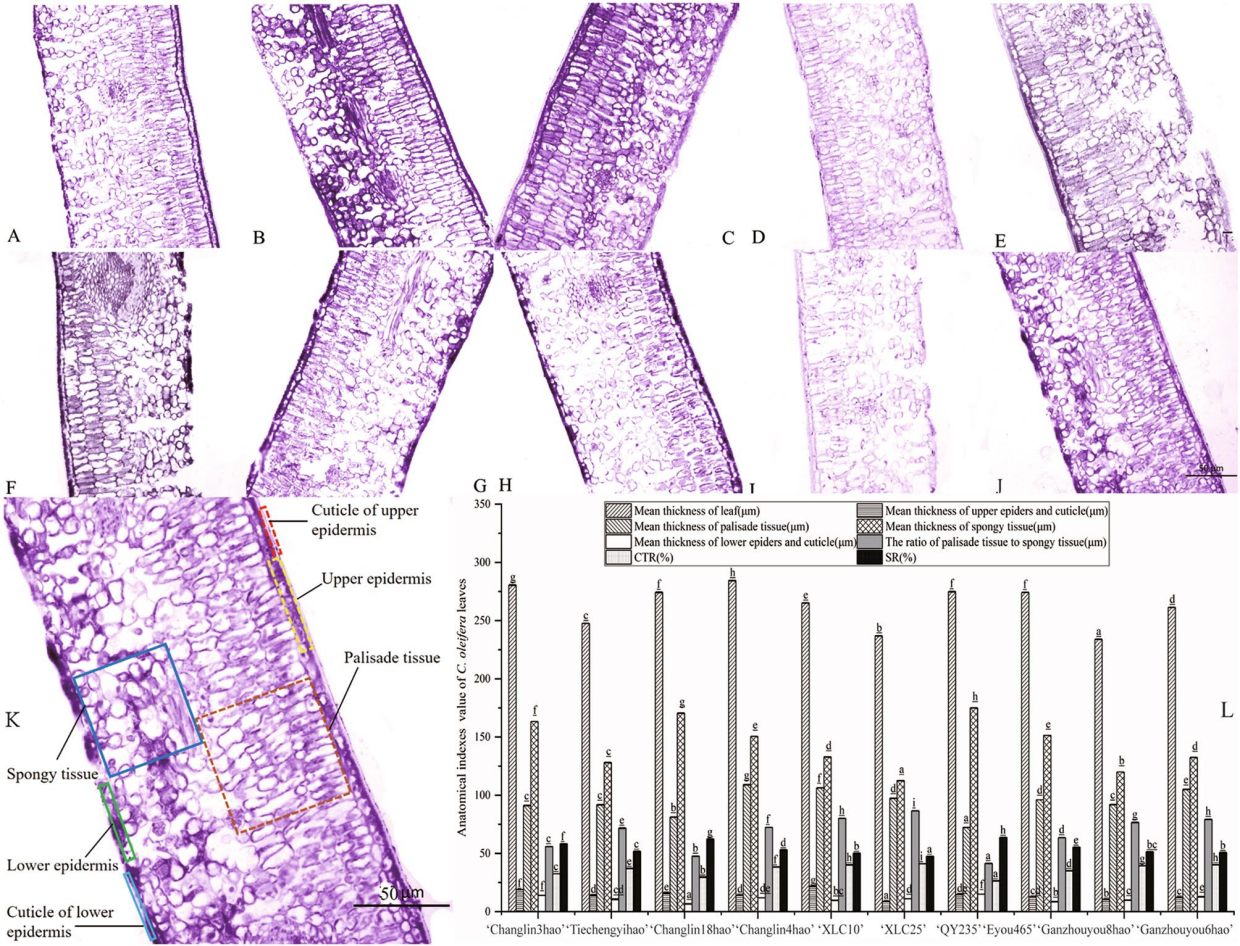
Leaf anatomical structure in 10 *C. oleifera* cultivars. (**A**–**J**) are pictures of ‘Changlin18hao’, ‘Changlin4hao’, ‘XLC10’, ‘XLC25’, ‘Changlin3hao’, ‘Tiechengyihao’, ‘Eyou465’, ‘QY235’, ‘Ganzhouyou8hao’, and ‘Ganzhouyou6hao’ leaf anatomy, respectively. K shows the values of different tissues of the 10 *C. oleifera* cultivars. Different letters within the same anatomical index in (**L**) indicate significant differences (P < 0.05) among the 10 *C. oleifera* cultivars. *CTR* closeness degree, *SR* looseness degree.

The leaf thickness of the 10 *C. oleifera* cultivars ranged from 233.86 to 284.31 μm, which included the upper epidermis (including the upper cuticle), palisade tissue, spongy tissue, and lower epidermis (including the lower cuticle) (Fig. 4). The thicknesses of the upper epidermis, palisade tissue, spongy tissue and lower epidermis of the 10 *C. oleifera* cultivars were 10.42–21.6 μm, 72.28–108.91 μm, 112.47–170.5 μm and 6.57–15.01 μm, respectively. The closeness degree (CTR) value of the cultivars ranged from 29 to 42%, and the looseness degree (SR) ranged from 47 to 63%.

### Correlation between cold resistance and leaf anatomical structure

Pearson correlation analysis revealed that cold resistance (LT_50_) was significantly positively correlated with leaf thickness and spongy tissue thickness (STT) and significantly negatively correlated with the palisade tissue, palisade tissue thickness (PTT)/STT and CTR (Table 4). A larger LT_50_-to-PTT ratio was associated with stronger cold resistance, and a larger LT_50_-to-SR ratio was associated with weaker cold resistance. Correlation analysis among the indices of the leaf anatomical structure showed a very significant positive correlation between leaf thickness and the spongy tissue, which indicates that the spongy tissue determined the thickness of the leaf. No other significant correlations were observed. The indices that were significantly negatively correlated with LT_50_ were included in the subsequent membership function analysis.

**Table 4:** Correlations among anatomical structure indices of *C. oleifera* leaves based on Pearson correlation analysis.

	LT	UECT	PTT	STT	LEC	PTT/STT	CTR	SR	LT_50_
LT	1								
UECT	0.581	1							
PTT	− 0.052	0.027	1						
STT	0.851**	0.459	− 0.560	1					
LECT	0.181	0.034	− 0.106	0.169	1				
PTT/STT	− 0.617	− 0.315	0.810**	− 0.935**	− 0.142	1			
CTR	− 0.558	− 0.280	0.857**	− 0.903**	− 0.175	0.992**	1		
SR	0.631	0.319	− 0.793*	0.944**	0.129	− 0.995**	− 0.984**	1	
LT50	0.672*	0.300	− 0.757*	0.938**	0.200	− 0.978**	− 0.977**	0.965**	1

In the table, LT, UECT, PTT, STT, LEC, PTT/STT, CTR, SR and LT_50_ represent leaf thickness, upper epidermis and cuticle thickness, palisade tissue thickness, spongy tissue thickness, lower epidermis and cuticle thickness, thickness ratio of palisade tissue to spongy tissue, closeness degree, looseness degree and semilethal low temperature, respectively. *Significant at (P = 0.05), **Significant at (P = 0.01). CTR, closeness degree. SR.

### Pollen structure

**Figure 5:**
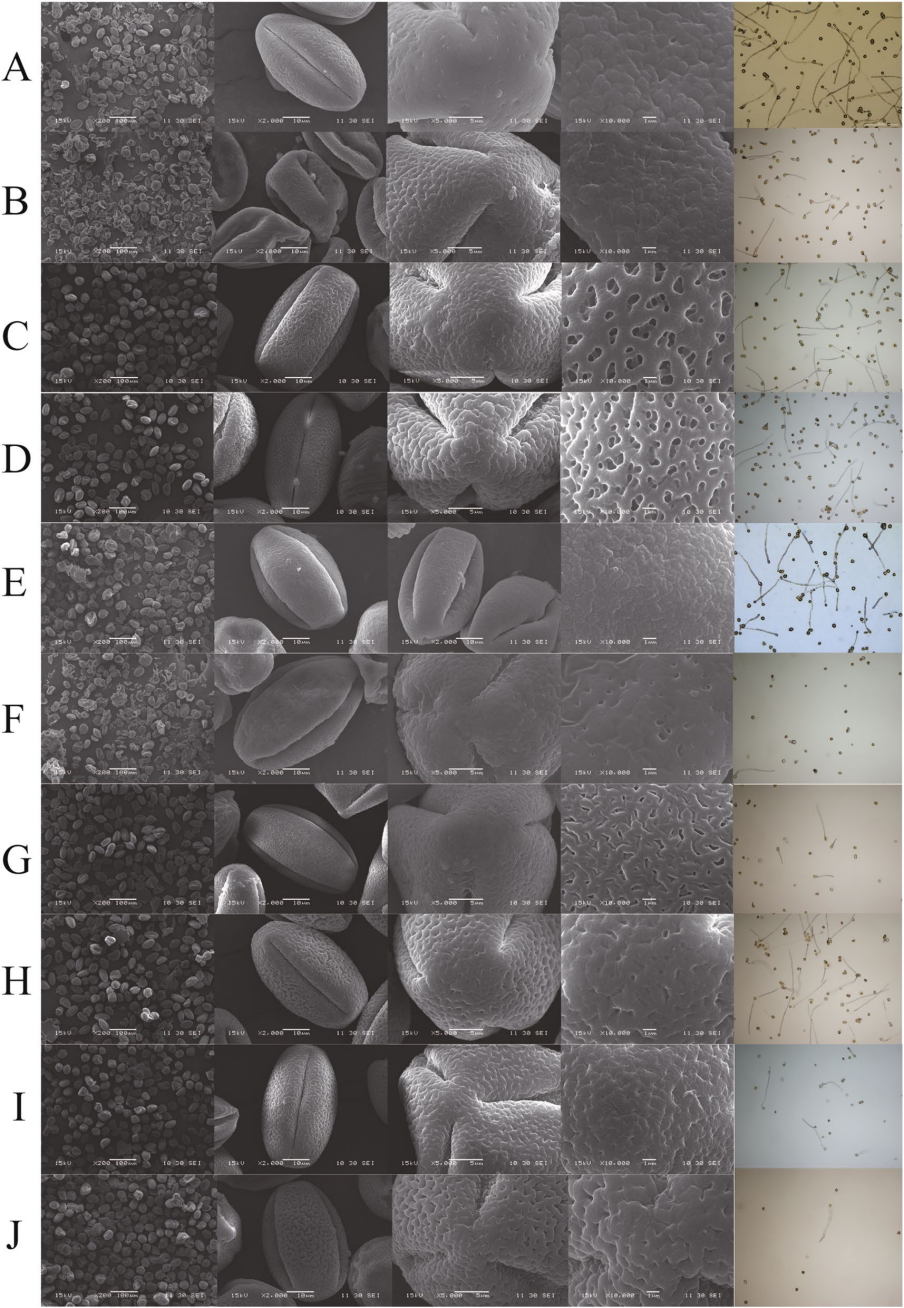
Morphology and viability of *C. oleifera* pollen. Figures (**A**–**J**) are ‘Changlin4hao’, ‘Changlin18hao’, ‘Ganzhouyou8hao’, ‘Ganzhouyou6hao’, ‘Tiechengyihao’, ‘Changlin3hao’, ‘XLC10’, ‘XLC25’, ‘Eyou465’, and ‘QY235’, respectively.

**Figure 6:**
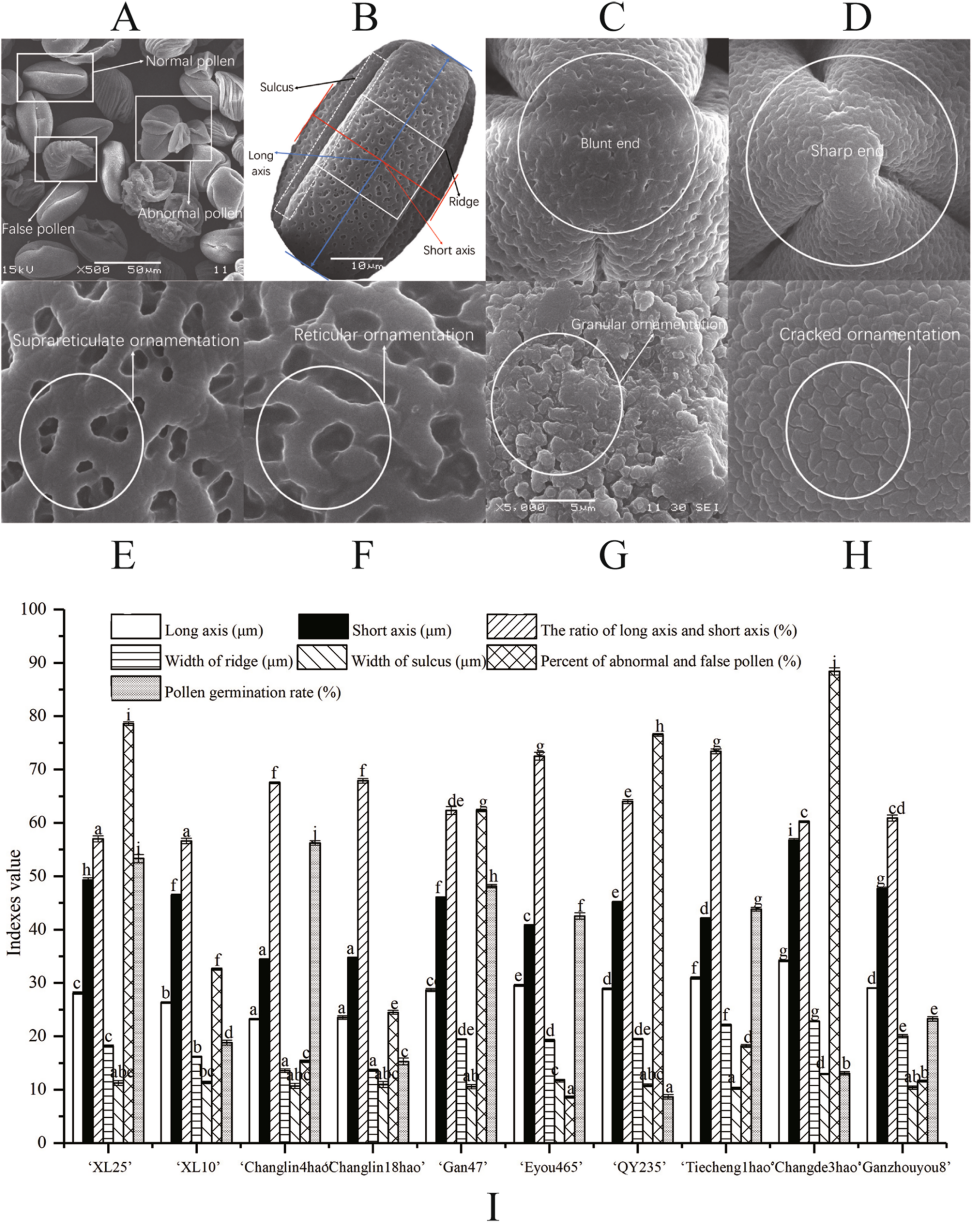
Phenotypic characteristics and index values of *C. oleifera* pollen. Figure (**A**) shows abnormal pollen and false pollen. Figure (**B**) shows the axis, sulcus and ridge of *C. oleifera* pollen. Figures (**C**) and (**D**) show the shapes of the end structures of *C. oleifera* pollen. Figures (**E**–**H**) show the external texture of *C. oleifera* pollen grains. Figure (**I**) shows the scanning index values and viability of *C. oleifera* pollen*.* Different letters within the same pollen index in (**G**) indicate significant differences (P < 0.05) among the 10 *C. oleifera* cultivars.

According to scanning electron microscopy (SEM), the pollen grains of the cultivars were monads, and they were tri-sulcate, ellipsoidal, isopolar, and bilaterally symmetrical (Figs. 5 and 6). In all 10 investigated cultivars, the sulcus extended from the distal end to the proximal end; it was narrower at the poles and wider at the equator, with sharp and rounded ends. The membrane ornamentations of the sulcus were highly variable and were reticulate, suprareticulate, cracked, and granulate. The reticula were consistently smaller toward the edge of the sulcus and larger at the lateral surface. Several remarkable differences in the dimensions of the pollen grains from the 10 cultivars were seen as well. The largest pollen grains were those of ‘Changde3hao,’ and the smallest were those of ‘Changlin18hao.’ The long axis of *C. oleifera* pollen grains ranged from 34 to 57 μm, and the short axis ranged from 23 to 35 μm, with a sulcus width of 10–13 μm.

### Pollen viability

Figure 6 shows the pollen viability of the 10 *C. oleifera* cultivars. The pollen viabilities of the 10 cultivars ranged from 8.67% to 56.23%, showing great variation.

### Correlation between pollen viability and cold resistance

The viability of *C. oleifera* pollen was not significantly correlated with the pollen structure, but it was negatively correlated with an abnormal pollen ratio (Table 5). Significant positive correlations were found among the indices of pollen structure. Additionally, the longitudinal diameter of pollen was correlated with the transverse diameter, ridge width, and longitudinal diameter/transverse diameter ratio. There was no significant correlation between the sulcus width and the transverse diameter of pollen, where the sulcus is seen as a series of ridges. This finding indicates that the sulcus width of *C. oleifera* pollen is generally constant and does not change according to the pollen parameters, such as ridge width (Table 5).

**Table 5:** Correlation among scanning indices of *C. oleifera* pollen according to Pearson correlation analysis.

	PLD	PTD	PAR	RW	SW	AFPR	PV
PLD	1						
PTD	0.795*	1					
PAR	− 0.651*	− 0.059	1				
RW	0.729*	0.974**	0.007	1			
SW	0.539	0.452	− 0.282	0.240	1		
AFPR	0.405	0.213	− 0.398	0.108	0.494	1	
PV	− 0.342	− 0.219	0.307	− 0.181	− 0.230	− 0.821**	1

In the table, PLD, PTD, PAR, RW, SW, AFPR and PV represent the longitudinal diameter, transverse diameter, aspect ratio, ridge width, sulcus width, abnormal and false rate, and viability of *C. oleifera* pollen, respectively. *Significant at *(*P = 0.05), **Significant at (P = 0.01).

### Correlation analysis of the main indicators

Possible correlations among the IBS (standardized value), stress (cold) resistance, pollen viability, LT_50_, PTT/STT, CTR, pollen viability, and pollen deformity ratio were analyzed. IBS was positively correlated with leaf cold resistance, leaf structure, pollen viability, PTT/STT, and CTR but negatively correlated with LT_50_ and the pollen deformity rate. The significant positive correlations of PTT/STT and CTR with pollen viability are indicative of the correlations among flowering time, pollen viability, leaf structure, and leaf cold resistance (Table 6).

**Table 6:** Correlation among different evaluation indices according to Pearson correlation analysis.

	FuBS	LT_50_	PTT/STT	CTR	PV	AFPR
FuBS	1					
LT50	− 0.703*	1				
PT/ST	0.650*	− 0.979**	1			
CTR	0.462	− 0.685*	0.706*	1		
PV	0.689*	− 0.568	0.639*	0.273	1	
AFPR	− 0.626	0.493	− 0.548	− 0.161	− 0.912**	1

In this table, FuBS, LT_50_, PTT/STT, CTR, PV and AFPR represent the full blossoming stage, semilethal low temperature, palisade tissue thickness/ spongy tissue thickness, closeness degree, viability of *C. oleifera* pollen and abnormal and false rate, respectively. *Significant at (P = 0.05), **Significant at (P = 0.01).

### Evaluation of C. oleifera cultivars by membership function analysis

The most important indices identified above were further used to determine the characteristics and advantages/disadvantages of the *C. oleifera* cultivars using membership function analysis (Table 7). The shorter the FuBS was, the earlier the plant blossomed. Pollen viability was also a positive index, whereas the pollen deformity rate, which was negatively related to pollen activity, was a negative index. The LT_50_ of *C. oleifera* was a negative index when the temperature fell below 0 °C but a positive index above 0 °C. PTT/STT and CTR were both positive indices. To calculate the overall membership value of a certain cultivar, the membership values calculated based on the negative indices were reduced by 1, and those calculated based on the positive indices were summed up. Then, the obtained result was divided by the total number of indices. A higher membership value indicates a greater potential for the given cultivar to grow in nonnative regions. Late-flowering *C. oleifera* cultivars such as ‘XLC25,’ ‘Changlin4hao,’ and ‘Ganzhouyou6hao’ had relatively higher membership values, whereas early-flowering cultivars such as ‘Changlin3hao,’ ‘Changlin18hao,’ and ‘QY235’ had relatively low membership values (Table 7).

**Table 7:** Evaluation of 10 *C. oleifera* cultivars with membership functions.

	FuBS	LT_50_	PTT/STT	CTR	PV	AFPR	Membership value	Order
‘XLC25’	0.907	1.000	1.000	1.000	0.940	0.880	0.955	1
‘Changlin4hao’	0.628	0.630	0.686	0.811	1.001	1.000	0.793	3
‘Ganzhouyou8hao’	1.000	0.863	0.839	0.936	0.831	0.916	0.898	2
‘Ganzhouyou6hao’	0.512	0.949	0.780	0.878	0.307	0.699	0.688	4
‘Tiechengyihao’	0.349	0.630	0.671	0.726	0.740	0.801	0.653	5
‘Eyou465’	0.326	0.497	0.490	0.590	0.713	0.962	0.596	6
‘XLC10’	0.140	0.897	0.856	0.932	0.214	0.123	0.527	7
‘Changlin3hao’	0.488	0.408	0.320	0.416	0.093	0.000	0.288	8
‘Changlin18hao’	0.163	0.240	0.139	0.221	0.139	0.327	0.205	9
‘QY235’	0.000	0.000	0.000	0.000	0.000	0.149	0.025	10

In this table, FuBS, LT_50_, PTT/STT, CTR, PV and AFPR represent the full blossoming stage, semilethal low temperature, palisade tissue thickness/spongy tissue thickness, closeness degree, viability of *C. oleifera* pollen and abnormal and false rate, respectively.

### Growth of the cultivars after transplanting

The results of the growth amounts of the cultivars after transplanting are summarized in Table 8. Two years after transplanting, the survival rates of all cultivars were above 90%, with an average of 92.4%. However, there were differences among cultivars from different origins. The survival rates of the two local cultivars in Hubei (i.e., ‘Eyou465’ and ‘QY235’) were both higher than the average, which indicated that the early-flowering and late-flowering local cultivars exhibited excellent adaptability. For the cultivars from other origins, the late-flowering cultivar showed a higher survival rate than the early-flowering cultivar from the same origin. These findings were consistent with the measured growth amounts of the 6-year plants: The growth amounts of the late-flowering cultivars were large, particularly ‘XLC25’ and ‘Changlin4hao’, whereas those of the early-flowering cultivars such as ‘Ganzhouyou8hao’ and ‘Changlin3hao’ were small. In addition, the overall difference in the growth amounts of the early-flowering cultivars between different years was much more noticeable than those of the late-flowering cultivars.

**Table 8:** The survival rates of 10 *Camellia oleifera* cultivars two years after transplanting, and the growth amounts of the six-year transplanted plants of different cultivars in 2018 and 2019.

Cultivars	Survival rate two years after transplanting (%)	Growth amount in 2018 (cm)			Growth amount in 2019 (cm)			Growth difference between years (cm)		
		TH	GD	CW (EWD/NSD)	TH	GD	CW (EWD/NSD)	TH	GD	CW (EWD/NSD)
‘Changlin4hao’	93.8b	7.7b	0.55a	7.8b/9.1b	6.7b	0.45b	7.3bc/8.8b	0.9c	0.09c	0.5bc/0.4c
‘Changlin18hao’	90.3e	6.6bc	0.46bc	6.7c/7.9d	5.1c	0.33d	6.1cd/7.2d	1.3a	0.12b	0.6b/0.6b
‘XLC25’	95.3a	9.1a	0.58a	8.6a/9.8a	8.2a	0.53a	8.3a/9.4a	1.0b	0.06d	0.3c/0.4c
‘XLC10’	90.5e	7.4b	0.42d	8.2ab/9.4ab	6.2b	0.36c	7.5b/8.6b	1.2ab	0.07d	0.6b/0.8a
‘Ganzhouyou6hao’	92.8c	5.9d	0.41d	6.3d/7.5e	4.9e	0.38c	6.0cd/7.1d	0.9c	0.04e	0.4c/0.3d
‘Ganzhouyou8hao’	92.6c	5.4e	0.47bc	6.5c/8.2d	4.2d	0.31d	5.8d/7.2d	1.3a	0.14b	0.7b/0.8a
‘Eyou465’	93.7b	7.3b	0.50b	7.9b/8.8c	5.6b	0.33d	7.1bc/8.2c	1.2ab	0.18a	0.8ab/0.6b
‘QY235’	92.5c	6.6bc	0.51a	7.6b/9.2b	4.8bc	0.28e	6.6c/8.1c	1.4a	0.20a	0.9a/0.9a
‘Tiechengyihao’	91.5d	6.1c	0.49b	6.6c/8.4cd	5.0c	0.39c	6.2cd/7.8cd	1.1b	0.08c	0.4c/0.5bc
‘Changlin3hao’	91.2d	5.6d	0.42d	6.2d/7.9d	4.3d	0.30d	5.4d/7.1d	1.3a	0.13b	0.7b/0.8a
Average	92.4	6.8	0.48	7.1/8.5	5.5	0.37	6.6/7.9	1.2	0.11	0.6/0.6

A different letter indicates a significant difference in the same column according to ANOVA corrected by the false recovery rate (FRD) method.

*TH* tree height, *GD* ground diameter, *CW* crown width, *EWD/NSD* east–west direction/north–south direction.

## Discussion

With the continuous changes in the climate and environment in recent years, plant ecospheres have changed year by year. The frequent occurrence of extreme weather events has posed huge challenges for plant growth. *Camellia oleifera* is an evergreen tree species that blooms in autumn and winter. In terms of vegetative growth, *C. oleifera* has strong cold resistance. However, in terms of reproductive growth, it is vulnerable to the impacts of low temperature. As an economic forest species, the primary purpose of growing *C. oleifera* is to obtain fruit. Therefore, the evaluation of *C. oleifera* introduction should be based on both vegetative growth and reproductive growth, which are different in different tree species.

Plant flowering phenology is regulated by complex factors. Although inheritance is the main regulator of phenology^8^, the external environment also has a strong influence. The external environment may cause plants to develop adaptive growth strategies and to evolve in the long term. However, to date, research on the flowering behaviors of *C. oleifera* has mainly focused on the morphological, physiological, and nutritional characteristics associated with flower-bud differentiation^9^. In this study, we found that some of the *C. oleifera* varieties introduced from different production areas had early flowering times, while some had late flowering times, which indicates that the flowering period of *C. oleifera* is determined by the joint actions of genetics and the external environment. The 10 varieties investigated in this study originated from different main production areas in China (Hunan, Zhejiang, Jiangxi, Hubei) with different genetic backgrounds. They were introduced at the Hubei Academy of Forestry and received the same management practices, but their flowering performance showed different characteristics. This finding indicates that genetic factors play a major role in the formation of the plant flowering period. In 2019, the research team also observed that all the investigated varieties showed late flowering due to high temperatures and drought. Nevertheless, the late-flowering cultivars showed later flowering times than the early-flowering cultivars. This finding indicates that the vegetative and reproductive growth of different *C. oleifera* cultivars exhibited varying degrees of adaptability to the external environment.

Plant stress resistance is closely related to its broader ability to adapt to the environment^10^. Recent evidence suggests the functional sequestration of leaf tissues, in which leaves are subjected to different levels of hydration during transpiration^11,12^. In this study, the PTT/STT ratio, SR and CTR were correlated with the cold resistance capability of *C. oleifera*. Canny et al.^13^ found that the palisade mesophyll maintains turgor during transpiration, whereas spongy mesophyll cells lose water much more readily than palisade matrix cells. P'yankov and Kondrachuk^14^ examined the leaf structure of 11 alpine plant species grown under natural conditions in the eastern Pamir Mountains at altitudes ranging from 3800 to 4750 m. According to them, changes in the mesophyll structure were associated with plant adaptations to mountain conditions, including differences in the number of cell layers and cell sizes in palisade tissue; the changes in these indices of mesophyll structure resulted in differences in leaf thickness and cell number per unit leaf area.

Pollen viability is influenced by climatic factors such as temperature and humidity^15^. In our study, the quantity of pollen collected in 2019 was relatively low, and the oval pollen vitality was lower than that reported in the literature^16^. This inconsistency is presumably due to the high temperature and drought in Wuhan in the summer of 2019. Heat stress can induce anther indehiscence, reduce pollen viability and reduce the proportion of ovules that receive a pollen grain^17^.

In this study, the phenotypic manifestations of different *C. oleifera* cultivars, such as the pollen size and surface textures, were quite different. This phenomenon is the result of long-term evolution under the influences of genetic and environmental factors. The pollen quantity and the viability of the early-flowering cultivars were generally lower than those of midseason-flowering cultivars, with significantly higher proportions of infertile and abnormal pollen grains (Fig. 6I). Presumably, this phenomenon is due to the greater sensitivity of male gametes in early-flowering cultivars to high temperature and droughts. Heat stress alters the chemical composition of pollen, slows pollen development, and detrimentally affects the shape of the pollen coat in the late stages of pollen formation^18^. For field pea^19^ and tomato (*Lycopersicon esculentum*)^20^, high temperatures control the lipid and protein composition and sugar content and can lead to reduced pollen viability. Prieu et al.^21^ reported that the rapid absorption of water by pollen grains risks pollen rupture; this change, in turn, is related to structural traits, such as aperture number, which controls the changes in pollen grain volume. McCallum and Chang^22^ studied the functional significance of pollen size, which is closely related to pollen tube length and growth rate, in *Ipomoea purpurea*. They found that the size of the individual pollen grains had a strong and positive effect on reproductive success.

In this study, the most important indices, such as FuBS, LT_50_, PTT/STT and CTR, were used to evaluate the 10 *C. oleifera* cultivars using membership function analysis (Table 7). Higher membership values were observed in late-flowering *C. oleifera* cultivars such as ‘XLC25’, and lower membership values were observed in early-flowering cultivars such as ‘Changlin3hao’. Therefore, late-flowering cultivars of *C. oleifera* had better cold tolerance than early-flowering cultivars. This finding indicates that late-flowering cultivars that are grown in the south have strong potential to be introduced to the north from the perspective of cold resistance. Nevertheless, detailed introduction procedures should also be developed based on a comprehensive consideration of the flowering and fruiting periods.

The results of the growth amounts of the 10 cultivars after transplanting showed that the growth amounts of the cultivars varied according to their origins. In the meantime, the early-flowering cultivars were more sensitive to adverse environment compared with the late-flowering cultivars. *Camellia oleifera* is an evergreen plant species, and it undergoes three shooting periods in one year. As spring shooting (from March to May) contributes the most to the nutritional growth of the tree, the growth amount of *C. oleifera* is likely to be influenced by its nutritional status in the previous year. In China, extreme weather often occurs in summer and autumn. In this study, 2017 was a normal year and 2018 was a dry year. Theoretically, the growth of *C. oleifera* in 2018 should be better than that in 2019. This theoretically-predicted result was supported by the measurement data in this study. Even more, the results of this study showed that the differences in the growth amounts of the early-flowering cultivars between different years were noticeably larger than those of the late-flowering cultivars. These findings indicate that the late-flowering cultivars have more excellent adaptability with more stable growth than the early-flowering cultivars.

The reproductive growth of *C. oleifera* spans four seasons and is easily affected by the external environment. In addition, it may also be influenced by precipitation and photoperiod. This study mainly analyzed indices of the organs of *C. oleifera*, such as cold resistance, flowering phenology and pollen activity, which do not completely reflect the stress resistance and growth characteristics of the different cultivars. Furthermore, phenotype formation, stress resistance and flowering in *C. oleifera* are closely related to hormones (such as gibberellins and abscisic acids) and key genes (such as *FT*, *SOC*, and *WRKY*). Therefore, studies on the associations of vegetative growth with reproductive growth, stress resistance and flowering from the perspectives of hormones and genes remain to be carried out in the future.

In conclusion, the *C. oleifera* cultivars, from high potential to low potential for introduction in the north, are ordered as follows: ‘XLC25’ > ‘Changlin4hao’ > ‘Ganzhouyou8hao’ > ‘Ganzhouyou6hao’ > ‘Tiechengyihao’ > ‘Eyou465’ > ‘XLC10’ > ‘Changlin3hao’ > ‘Changlin18hao’ > ‘QY235’. The results of this study may be significant in guiding not only the industrial planning of *C. oleifera* production in China but also the introduction and domestication of other plants worldwide.

## Material and methods

### Plant materials

The *C. oleifera* trees examined in this study were grown in the *C. oleifera* germplasm resource repositories of the Hubei Academy of Forestry (Jiufeng Mountain, Wuhan, Hubei Province, 113° 41′–115° 05′ E, 29° 58′–31° 22′ N) under natural conditions. They included nationally or locally approved cultivars with different growth habits and origins (Table 1). The trees were originally collected from the main cultivation areas (original regions) in China during a single year, and their age ranged from 6 to 8 years. The investigated area experiences a subtropical monsoon humid climate with an absolute highest temperature of 42 °C and an absolute lowest temperature of − 16 °C. The lowest average temperature in a year (3.0 °C) occurs in January, and the highest average temperature (29 °C) occurs in July. The summer lasts 135 days. According to the statistical data released by the Wuhan Meteorological Station (30° 37′ N, 114° 80′ E), the annual average precipitation is 1205 mm, and the annual average evaporation is 1391 mm, with a relative humidity of 77%; the accumulated temperature is between 5000 and 5300 °C, and the annual frost-free period lasts 240 days. For the purpose of the experiment, the trees were not irrigated in summer and autumn in 2019, with other management following routine practices. The soil is a yellow–brown soil, and the primary parent materials include hydromica, vermiculite and kaolinite; the soil had a pH value of 5.43–5.97. In the 0–20 cm soil layer, the total nitrogen content was 0.14%, the carbon–nitrogen ratio was 17, the free iron oxide content was 2.96%, the exchangeable acid content was 3–6 mg equivalent/100 g, the exchangeable aluminum content was lower than 0.3 mg equivalent/100 g, and the base saturation percentage was 52–94%.

### Tree cultivars and their studied properties

The flowering time, cold resistance, phenotype indices, pollen morphological indices, pollen viability, and growth after transplanting were investigated in 10 *C. oleifera* cultivars introduced from four producing areas: ‘QY235,’ ‘Changlin4hao,’ ‘Tiechengyihao,’ ‘Eyou465,’ ‘Ganzhouyou8hao,’ ‘Ganzhouyou6hao,’ ‘XLC25,’ ‘XLC10,’ ‘Changlin3hao,’ and ‘Changlin18hao’ (Table 9). These cultivars are representative of the early- and late-flowering cultivars in the producing areas. Their growth ranges are extensive, and they show great application potential.

**Table 9:** *C. oleifera* cultivars analyzed for multiple-index relationships.

Original region	Cultivar	FuBT	Original region	Cultivar	FuBT
Hunan	‘XLC25’	40	Zhejiang	‘Changlin4hao’	28
	‘XLC10’	7		‘Changlin18hao’	8
Hubei	‘Eyou465’	15	Jiangxi	‘Ganzhouyou8hao’	44
	‘QY235’	1		‘Ganzhouyou6hao’	23
Hunan	‘Tiechengyihao’	16	Hunan	‘Changlin3hao’	22

*FuBT* full blossoming time.

### Flowering time

At least five individual trees of each cultivar were continuously observed at the Hubei Academy of Forestry in 2016 and 2017, from before the bloom period in late September to the final blooming stage. Based on the accepted criteria for fruit trees, florescence phenology can be divided into the IBS (5–25% of flowers open), the FuBS (25–75% of flowers open), and the FiBS (> 75% of flowers open)^7^. To facilitate the analysis, the date of each flowering stage was standardized (Table 1, Columns 7 to 9) before the data were used in a Pearson correlation analysis to determine the flowering stage that is the most important for the growth of *C. oleifera*. Standardization was performed as follows. The blossoming stages, i.e., IBS, FuBS and FiBS (recorded in Table 2), were obtained from the corresponding dates for each cultivar (Table 1) by subtracting the corresponding dates for the earliest cultivar (‘QY235’), i.e., October 14, October 18, and October 31 (shown in bold in Table 1, Columns 4 to 6 of the third row). The obtained stage data were then used for a subsequent multi-index correlation analysis. The 22 *C. oleifera* cultivars were clustered based on the three blossoming stages (IBS, FuBS, and FiBS) and the FuBSDT. The data were subjected to hierarchical cluster analysis (a statistical method used in the construction of a dendrogram) in SPSS. The dendrogram was constructed based on the average linkage between groups, using the Euclidean distance as a similarity index^23^.

### Cold tolerance

Fifteen branches with current-year leaves were collected from three *C. oleifera* trees in December 2018. Five branches in the same tree were considered a biological replication. After a low-temperature gradient treatment, the leaves of the five branches were evenly mixed and divided into 3 groups. For each group, the physiological indices of cold resistance were measured three times. Moisture-retaining branches were transported to the laboratory for immediate low-temperature gradient stress testing in freezer boxes at temperatures of 0 °C, − 5 °C, − 10 °C, − 15 °C, and − 20 °C. The freezer boxes were cooled in a programmable freezer (HX-010; Planer, Inc., Sunbury on Thames, UK) at a rate of 0.1 °C/min from 4 °C to the designated temperature. After 24 h away from light, the branches were removed and allowed to thaw at 4 °C for 3 h and then at room temperature for 12 h to determine the LT_50_ based on ion leakage with a conductometer (Zhuoer DDG-5101, Anhui, China), according to a previously described method^24^. The percentage of ion leakage was calculated as [conductivity of the leachate after freezing stress-conductivity of nonfrozen controls] × 100/[final conductance after killing-conductivity of nonfrozen controls]. The leaf soluble protein was determined using the protein dye-binding method^25^, with bovine serum albumin as the standard. Free proline was estimated using spectrophotometric (Leica Camera AG, Germany) analysis at 515 nm of the ninhydrin reaction^26^. Malondialdehyde was determined based on thiobarbiturate reactions^27^.

### Leaf structure

Leaves that had germinated in spring were collected in December 2018 for tissue analysis. Four leaves were removed from each of the three plants of the same *C. oleifera* cultivar. A 5 × 5 mm square was cut off from each leaf (the midrib was avoided) and then fixed in formaldehyde/acetic acid/ethanol/water (2/2/27/9, v/v/v/v). Afterwards, the samples were dehydrated in a graded series of ethanol solutions followed by paraffin embedding^28^. Approximately 8–10-μm sections were made with an RM 2265 microtome (Leica Camera AG, Wetzlar, Germany). The sections were stained with Safranin and Fast Green according to Sass’s method^29^. Images of different cross-sections were taken under a microscope (BX-51; Olympus, Tokyo, Japan) and processed using ImageJ software (ImageJ 1.47v; National Institutes of Health, USA). The thicknesses of the spongy and palisade tissues and of the upper and lower epidermis, including the cuticle, were measured based on 20 replicates per tissue and per image. The CTR and the SR of the organizational structures were calculated as follows^30^:

$${\text{CTR }} = \, \left( {{\text{PTT}}/{\text{leaf }}\;{\text{thickness}}} \right) \times { 1}00\% ;$$

$${\text{SR }} = \, ({\text{STT}}/{\text{leaf }}\;{\text{thickness}}) \, \times { 1}00\%$$

### Pollen viability

Pollen viability was assessed using a tetrazolium test. Anthers were collected from three trees of each cultivar for the test. One flower bud in each direction (east, west, north and south) of the central periphery of the *C. oleifera* crown was collected from each tree. The pollen grains from one tree were considered a biological replicate. One culture dish was prepared for each replicate, and in each culture dish, three visual fields were observed. The number of pollen grains in each visual field was kept at less than 20. The collected flower buds were about to open, and in each cultivar, they grew normally and synchronously with a uniform-size cultivar. The collected anthers were placed in Kraft paper bags and immediately transported to the laboratory, where they were dried at − 30 °C until the pollen was released. The pollen was then cultivated in 100 mL of medium (100 g sucrose/L, 100 ppm H_3_BO_3_ and 300 ppm Ca(NO_3_)_2_ 4H_2_O, and distilled water) at 30 °C for 12 h. Then, the growth of the pollen tube was observed using microscopy (BX-51; Olympus, Tokyo, Japan).

### Pollen structure

The morphological features of the pollen grains were observed using SEM (Jeol 100 CXII). The method for sampling was the same as that performed in the experiment on pollen vitality. The pollen grains from the same cultivar were mixed. For each cultivar, 3 visual fields were observed (× 2000), and in each field, 5 pollen grains were measured (the pollen grains were well arranged for ease of measurement). The pollen grains were platinized with an ion sputtering equipment (ETD-2000) for SEM, and no less than 20 pollen grains for each cultivar were examined. The shriveled pollen grains with great deformities under the scanning electron microscope were defined deformed. Under the microscope (× 200), five visual fields were randomly selected, and no less than 100 pollen grains in each field were observed.

### Growth after transplanting

Survival rates of the saplings of different *C. oleifera* cultivars were calculated two years after transplanting. The criteria for survival after transplanting were that the sapling survived and did not show noticeable differences compared with those at the same age which grew in the original area. For each cultivar, three sample plots (approximately 300 m^2^ for each) were randomly demarcated. The spacing of the plants was 2 m × 3 m, with no less than 50 plants in each plot. Plants in normal growth that were aged six years after transplanting were used to compare the growth amounts of different *C. oleifera* cultivars. For each cultivar, 12 plants were randomly measured, with 4 as a replicate. *Camellia oleifera* is an evergreen tree species, and it shoots three times in one year, which occurs in spring (from March to May), summer (from May to August) and autumn (September to November), respectively. Therefore, the height, ground diameter and crown width of the plant was measured in February and in December, respectively. The growth amount in a year was calculated by subtracting the measurements obtained in February from those obtained in December of the same year. The ground diameter of a plant referred to its trunk thickness 5 cm above the ground, which was measured with a vernier caliper. The height and crown width were measured with a band tape. The crown width of the tree was presented as the maximum values in the east–west and south-north directions. The year 2017 was a normal year and 2018 was a dry year. The differences in the growth amounts of the early-flowering cultivars and late-flowering cultivars between 2018 and 2019 were compared.

### Statistical analysis

Numerical data are presented as the mean ± standard deviation (M ± SD) and were analyzed with SPSS 22.0 (IBM Corp., Armonk, NY, USA). Data satisfying a normal distribution were analyzed using one-way ANOVA adjusted by the false discovery rate (FDR). Otherwise, nonparametric Kruskal–Wallis ANOVA was performed. P < 0.05 was considered statistically significant. Origin (ver. 8.0; Origin Lab Corp., Northampton, MA, USA) and Adobe Illustrator (ver. CS4; Adobe Corp., San Jose, CA, USA) were used for data mapping and image processing. Pearson correlation analysis was performed to determine the leaf anatomical structure-related index that was most closely associated with cold resistance and the key pollen-related index for subsequent membership function analysis.
